# Making student mistakes didactically useful in bedside teaching: relevance of learning content, teacher and patient presence

**DOI:** 10.1186/s12909-026-09978-y

**Published:** 2026-07-28

**Authors:** Hannah P. K. Rubisch, Anna-Lena Blaschke, Pascal O Berberat, Martin Gartmeier

**Affiliations:** TUM School of Medicine and Health TUM Medical Education Center, Technical University of Munich (TUM), Ismaninger Straße 22, Munich, 81675 Germany

**Keywords:** Bedside teaching, Medical education, Student mistakes, Teacher reactions, Teaching methods, Teacher presence, Patient presence, Video study

## Abstract

**Background:**

Student mistakes are common in bedside teaching (BST) and can serve as important learning opportunities. However, little is known about how frequently mistakes occur in authentic clinical settings, what types of learning content they involve, and how teacher and patient presence shape teachers’ handling of these mistakes. This study investigated (RQ1) the relevance of learning content during student mistakes, (RQ2) associations between mistake types and teachers’ reactions, and (RQ3) whether patient presence influences clinical teachers’ responses to mistakes.

**Methods:**

We conducted an observational study based on video recordings of 36 BST sessions across internal medicine, neurology, and orthopaedics (78.4 h). Using a hierarchical coding scheme, we identified mistakes, classified their learning content and mistake types, and coded teacher reactions as well as teacher and patient presence. Interrater reliability ranged from substantial to excellent. Descriptive statistics were used to determine the frequency of mistakes per hour and their distribution across the learning content. Associations between mistake types and teacher reactions were analysed via Spearman’s rank correlations (RQ2). Mann–Whitney U tests (RQ3) were used when examining whether teachers’ mistake-related behaviours differed depending on the presence or absence of the patient.

**Results:**

Across all BST sessions, the students made an average of 4.9 mistakes per hour. Mistakes occurred most often during theoretical knowledge and clinical reasoning, predominantly as reproduction mistakes, whereas an incorrect application of skills was most frequent during clinical examination and case presentations. Teacher presence was positively associated with the number of observed mistakes, particularly reproduction mistakes. During the physical examination, the students were supervised for only 72% of the active practice time. Teachers’ reactions - including elaboration, directness of feedback, and time provided for correction - did not differ significantly depending on patient presence.

**Conclusions:**

Student mistakes are common in BST and hold considerable didactic value; however, this value is not fully realised when supervision is inconsistent or when feedback remains limited. Enhancing clinical teachers’ capacity to detect errors, providing structured and supportive feedback, and adapting their approach to the presence of patients may strengthen their ability to learn from mistakes. Interventions that cultivate psychological safety and promote effective error-management practices could further improve the pedagogical quality of BST.

## Background

Bedside teaching (BST) is a traditional, interactive clinical teaching format and an essential component of medical training. It is advanced that instruction at the bedside is “rich with visual, auditory and tactile experience” [1]. In its classic form, BST mainly includes teaching in the presence of a patient [2], providing students with the opportunity to practice essential clinical skills such as communication, medical history taking, clinical reasoning, physical examination and diagnosing of real patients, (ideally) under the supervision of clinical teachers [3].

However, more modern forms of BST also include off-patient activities, such as lecturer-guided discussions of patient cases. Rousseau et al. noted that physicians and students “value teaching and learning of physical examination skills, with multiple hands-on opportunities for direct role modelling, coaching, observation and deliberate practice” [4]. Therefore, BST can be considered didactically valuable, in addition to other aspects, because it offers very good opportunities to apply clinical *skills* (especially with respect to examination and communication) and clinically relevant *knowledge* (in clinical reasoning) to specific patient cases. Our study focuses on an often-overlooked aspect of BST: its potential to facilitate learning from mistakes. Because BST requires students to apply diverse skills and knowledge, errors naturally occur; however, the didactic value of these errors depends heavily on how clinical teachers manage them.

Despite subst antial research on didactical aspects of BST [5–21], whether and how student mistakes are handled in didactically fruitful ways by clinical teachers in BST has rarely been investigated. To fill this gap, we aim to describe clinical teachers’ practices of handling student mistakes at the bedside in the current study. For this purpose, we video-recorded bedside teaching sessions in clinical medical education in the subject areas of internal medicine, orthopaedics and neurology and, on this basis, conducted fine-grained analyses of the recorded interactions. In a first paper based on these data [22], we addressed the following questions: which types of student mistakes can be observed in BST, how do clinical teachers react to these mistakes, and do they use different strategies to address different types of mistakes? These points are important, as present-day medical education on BST faces significant challenges, e.g., regarding the quantity of BST being in decline [1, 4, 5, 23–28]. The decline in BST is driven by multiple, interrelated challenges. Increasing time pressures from high patient volume and the prioritisation of clinical work over teaching limit opportunities for bedside instruction [4, 6, 29]. Curriculum design and larger student groups reduce individualised attention, while clinical teachers often face inadequate preparation, low motivation, and limited institutional support [4, 11, 20, 30, 31]. Student-related factors, including insecurity, fear of embarrassment, and gaps in foundational knowledge, further hinder active engagement [4, 6, 32]. Finally, patient concerns, shorter hospital stays, and reliance on electronic records and diagnostic technologies have diminished the role of the bedside as a central site for clinical education [4, 6, 28, 33]. This trend underlines the relevance of focusing aspects of the didactic quality of BST [26], such as the way student mistakes are fruitfully used for didactic purposes by clinical teachers.

The social structures of BST are increasingly heterogeneous, meaning clinical teachers cannot always supervise the entire student group simultaneously. Often, groups split to interact with different patients, leaving some students entirely unsupervised [4]. We hypothesise that such situations are disadvantageous with respect to learning from mistakes because without the lecturer being present, mistakes might occur unnoticed and, consequently, are not used didactically.

This would be disadvantageous, as many researchers argue that student mistakes can be didactically very useful, as they allow clinical teachers to gain insight into gaps or misconceptions in students’ knowledge [34, 35]. Analysing student mistakes is hence a promising perspective for medical education research, as it helps to better understand how learning from mistakes in BST can be fostered. This, in turn, may contribute to decreasing the frequency of errors in students’ later everyday clinical work [36]. On the basis of information about what students do not (yet) know, where their clinical understanding is limited, or about which skills students have not yet mastered, clinical teachers can initiate learning activities specifically tailored to improve students’ knowledge and skills in very targeted, situation-adequate ways [34, 37–40]. Moreover, learning from mistakes can foster the development of *negative knowledge*, i.e., knowledge about what assumptions are *incorrect* or about how something does *not* work. It has been argued that such knowledge is valuable in professional contexts, as it provides a cognitive resource for avoiding mistakes based on previous, error-related learning processes [41]. BST offers very promising opportunities in this respect, as it is highly interactive and occurs mostly in small-group settings, in which clinical cases are discussed in detail [42].

To shed further light on what are good ways for clinical teachers to make didactic use of student mistakes, the current study focuses on the relationship between the contextual conditions and the occurrence of student mistakes in BST. This is a hitherto neglected but important focus, as knowledge of the contextual conditions in BST, which make the occurrence of student mistakes more likely, is relevant for clinical teachers. This is because it can assist clinical teachers in planning BST sessions accordingly and in staying particularly vigilant during phases in which they can expect to observe and make didactic use of student mistakes. More specifically, our research questions (RQs) concern in which types of learning content student mistakes can be observed and how the presence of clinical teachers and patients influence the occurrence of student mistakes.

### Conception of mistakes in BST

In defining what constitutes a mistake, we build on our previous work [22], and follow the definition of Bauer [43]: In this understanding, labelling an action or its outcomes as *mistake* indicates a deviation from a specific goal or standard - such as when a student elicits the patellar reflex with an inadequate tap and incorrectly reports it as normal. Further, we follow the argumentation advanced by Heid [44], who hints towards the relevance of hierarchy in social structures for the identification of actions as erroneous. In this view, the judgement of a phenomenon as a mistake is connected to an authoritative process, whereby hierarchically superior individuals play a primary role in defining what counts as an error in a specific context. On this basis, we define (and empirically capture) mistakes in BST as instances where another individual (either a clinical teacher - or possibly also a medical student) addresses a mistake by means of making an explicit verbal remark. Such remarks can be either knowledge-related (what a student *said*) or skills-related (what a student *did*). More specifically, knowledge-related mistakes encompass reproduction (missing or incorrect facts), comprehension (flawed connections), application (incorrect use of facts in a patient case), and analysis (flawed clinical reasoning). Skills-related mistakes primarily involve the incorrect procedural application of a medical skill (cf. Table 1 [22, 45]), .

### Learning from mistakes and learning content covered in BST

With respect to learning content, the main learning objectives of BST discussed in the pertinent medical education literature are history taking, physical examination, case presentation, clinical reasoning and theoretical knowledge [14, 15, 46]. As discussed above, the bedside is also ideal for teaching further learning objectives, such as professional behaviour, humanism and respectful physician‒patient interactions [7, 23]. We hypothesise that these learning objectives differ in terms of the opportunities for learning from mistakes they afford, on the one hand, because, in the context of some learning objectives, more mistakes occur than in others; on the other hand, different types of mistakes might occur more or less frequently in the context of different learning objectives. To address these questions, we examine the interrelation between the learning content covered in BST, on the one hand, and the frequency and type of student mistakes that occur, on the other hand.

### Learning from mistakes and teacher presence in clinical examinations

Furthermore, we investigate the relationship between the presence or absence of a clinical teacher and the handling of student mistakes, whereby we focus on the learning content of a *clinical examination*. The focus on teacher presence is relevant because the way teachers react to and handle mistakes strongly influences the degree to which mistakes can be learned from [47]. To properly contextualise our data, it is important to note that in German undergraduate medical education, BST refers to an entire curricular module, rather than just the isolated moments of direct clinical instruction. Aligning with comparable curriculum designs in this context [13], these sessions typically span approximately 150 to 180 min and feature a combination of theoretical and practical components. The format frequently alternates between instruction in the full group and practical application of clinical skills in smaller sub-groups. Consequently, phases of independent student practice at the bedside - while the clinical teacher is rotating between other rooms - are structurally embedded in this form of BST. Because our goal was to investigate the entire scheduled format, we analysed the full duration of these sessions, deliberately including mistakes that occur during these designated, yet unsupervised, practice phases.

When learning clinical examinations in the context of BST, medical students acquire skills through practical training. They often start by examining fellow students until they gain a deeper understanding of specific examination techniques and their anatomical/physiological background. They subsequently practice with patients who were previously assessed by doctors within supervised clinical training. In this study, we focus on clinical examination as learning content. This is because in the context of our previous study [22], we observed a large variance in teacher presence vs. absence as well as frequent student mistakes during clinical examination. Furthermore, the practical nature of clinical examination and its multistep character provide a good basis for observing errors in the presence vs. absence of medical teachers, who did often rotate between student groups.

### Learning from mistakes and patient presence

Eventually, we investigate how patient presence influences the occurrence of student mistakes in BST. As discussed above, mistakes may give direction to the learning process - possibly more than tasks that were completed successfully. However, despite their learning potential, mistakes are rarely utilized effectively in teaching [40]. In medical education, a pervasive “stigma of being wrong” often exists. Removing this stigma is essential to creating a supportive environment that encourages critical thinking, open expression, and timely feedback, even in the presence of patients [48]. In this respect, the presence of a patient as a relevant third party is accentuated as a specificity of medical education in general and of BST in particular. Students may feel embarrassed in front of a patient, particularly when they have made a mistake. Furthermore, such situations may also affect the doctor-patient relationship: Williams et al. described concerns expressed by medical teachers during focus group discussions related to the notion that mistakes occurring during bedside teaching could cause patients to lose confidence in the students as clinicians and in the teachers as team leaders [23]. “It is likely that doctors are aware of this situation and adjust their response in front of the patient. Further research on […] how the teacher’s reaction changes in the presence and absence of the patient is needed” [22].

In summary, we examine differences in the occurrence of student mistakes (as an important prerequisite for learning from these mistakes) depending on the social form (plenary vs. small group, teacher present vs. absent, patient present vs. absent). We focus the following RQs in the present study:

- RQ 1: How is the learning content covered in bedside teaching related to the frequency and types of student mistakes which are addressed, either by clinical teachers or students?
- RQ 2: How does the presence or absence of medical teachers during a clinical examination impact the frequency and types of mistakes that are addressed?
- RQ 3: How do clinical teachers’ reactions to student mistakes change depending upon the presence and absence of the patient during BST sessions?

## Methods

We report a single-centre, observational study using video data from 36 bedside teaching sessions. Videos were coded based on a literature-informed hierarchical framework, allowing for quantitative description of low-inferential features such as learning content, presence of teaching physicians and patients, and observed students’ mistakes. In a two-step process these mistakes were subsequently characterised for teacher reaction and type of mistake.

### Bedside teaching sessions

The BSTs analysed in this study were part of the structured curriculum for undergraduate medical students at the Technical University of Munich. These sessions built on first-year peer teaching, combined patient contact with seminar-style learning, and prepared students for block placements in the third clinical year. Students receive a total of twelve BSTs in their second clinical year (fourth year of medical studies overall), of which eight were internal medicine, two were neurology, and two were orthopaedics, each scheduled for 180 min. Each time, they are placed in a different medical speciality and tutored by a physician exempted from ward duties. We videotaped 39 BST sessions in clinical medical education at TUM Rechts der Isar University Hospital. Each course was attended by approximately 6 students. In the local medical curriculum, a total of 360 such sessions are offered each semester over a time span of twelve weeks.

### Sample, recruitment and ethical considerations

In a pilot-study, we filmed three BST sessions. These data were used to examine and optimize the process of data collection, particularly the coding scheme developed for the subsequent main study. In the main study, a total of 36 BST sessions were videotaped, 12 in internal medicine, neurology, and orthopaedics, respectively. All analyses reported in this paper rely on data from these sessions. The selection of BSTs to be filmed took place at least two weeks prior to the respective session. The teaching clinician was contacted one week before the recording to discuss open questions. The clinical teacher identified suitable patients on the ward and asked for their consent to participate in both the BST session and the study. We obtained written approval for participation in the study from all patients, students and clinical teachers. The students in these sessions received an e-mail in advance and were given the opportunity to change to another course if they did not wish to take part in the study. Seven students switched sessions.

The 36 videotaped BST sessions had a cumulative length of 78.4 h. They were taught by 24 different teachers. This means that we filmed twelve teachers once and twelve teachers twice. A total of 259 students and 84 patients took part in the filmed sessions. Out of the latter, 47 were audible (but not visible) in our video data, and the others were visited by subgroups of students not accompanied by a cameraperson from our research team. At the beginning of each session, before starting the recording, a member of our team was present to again inform the participants about our study, respond to arising questions and collect written consent from all participants. We obtained full consent prior to any filming. The ethics committee of the TUM rechts der Isar University Hospital reviewed and approved the present study (Ethics vote code: 360/18S).

### Video recording of bedside teaching sessions

**Fig. 1:**
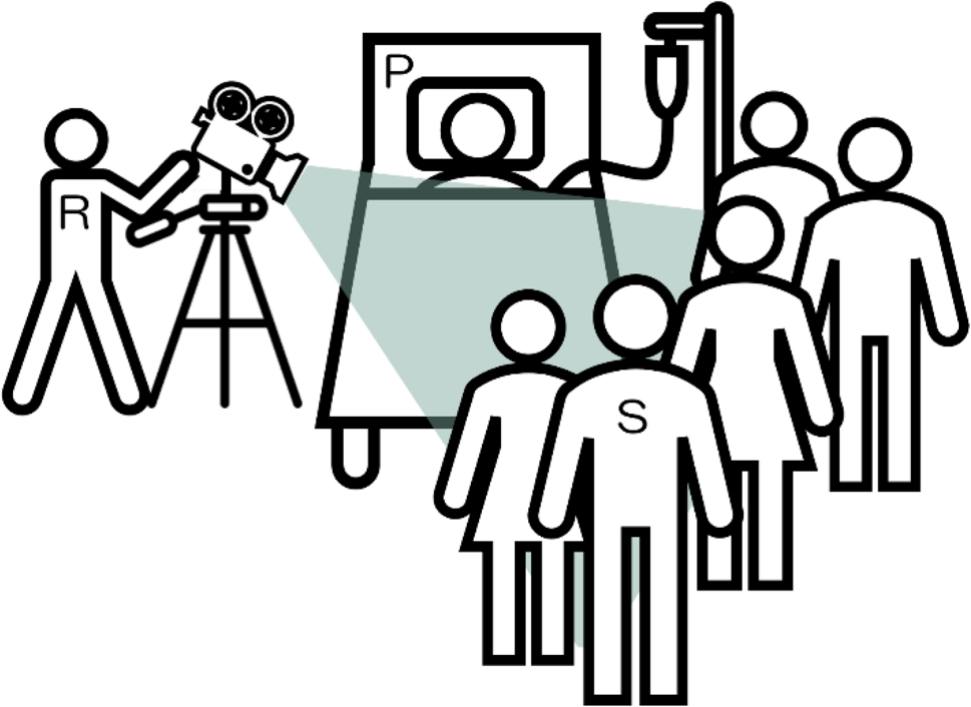
Camera setup during video recording in the patient room; R = researcher; P = patient; S = students

As illustrated in Fig. 1 [49], we used a video camera with a tripod and directional microphone to record the BST sessions. We did not pause or stop the recording until the clinical teacher had finished the session to ensure comparability of the videos. The camera followed the group when the teaching location was changed (e.g., from a meeting room to a patient room). In general, we filmed from an overview perspective so that the teacher, the students and, where appropriate, other (e.g., medical) objects were recorded. In the patient room, we ensured that the faces of the patients were not visible in the videos to retain their anonymity. To do so, the cameraperson always stood next to the head of the patient’s bed, and the camera was directed towards the group of students and the lecturer. In this way, the patients were not visible but were audible in the video recordings. At the beginning of each session, the cameraperson silently chose one student in the group at random whom the camera followed, e.g., in cases when the group was divided into smaller groups. This means that if different patients were examined simultaneously, the camera did not switch between subgroups but remained with the randomly selected students’ subgroup. Overall, the cameraperson tried not to influence the course of the session as far as possible.

### Analysing the video material: categorial system and coding

To analyse the video data, we created an exhaustive-disjunctive^1^ hierarchical categorial system on the basis of the rating schemes developed by Seidel et al. [49, 50]. We first used the video data from the preliminary study to train the raters: both raters coded video material from the preliminary study and then visualized and compared the results and discussed deviations. Through an iterative process, initially defined categories were revised multiple times to further refine our categorical system. Only after a satisfactory level of agreement was reached did we move on to the main study. Interrater reliability was determined based on 37,418 one-second intervals generated after coding four videos (total duration: approximately ten hours).

Two members of our research team (AB and HR) used *Microsoft Excel* and *Mangold Interact* to code the video data regarding *student mistakes*, *learning content* and *social structure* [49].

In the first round, all *student mistakes* were marked along with other codes. Consistent with our operational definition, we assigned the code *mistake* on the basis of the occurrence of an explicit verbal correction made by the medical teacher or by a fellow student in the respective course group [51]. Mistakes on both verbal and nonverbal levels (e.g., during the clinical examination) were included. In a subsequent step, we differentiated several types of mistakes on the basis of the classification advanced by Anderson and Krathwohl [45] on the basis of Bloom’s taxonomy ([52], cf. Table 1 [22]), . In a further coding round, we operationalized teacher reactions by drawing upon three categories adopted from Wuttke [53, 54] and Crespo [55], i.e., *feedback*, *elaboration* and *time for correction* (cf. Table 2 [22]), .

Regarding the reliability of the measurements, we found a high interrater agreement value of Cohen’s Kappa = 0.84 for both categories, *student mistakes* and *types of student mistakes*. For *feedback*, a satisfactory value of Cohen’s Kappa = 0.64 was reached. For the *level of elaboration* and *time for correction*, the values were Cohen’s Kappa = 0.91 (very good) and 0.66 (satisfactory), respectively.

**Fig. 2:**
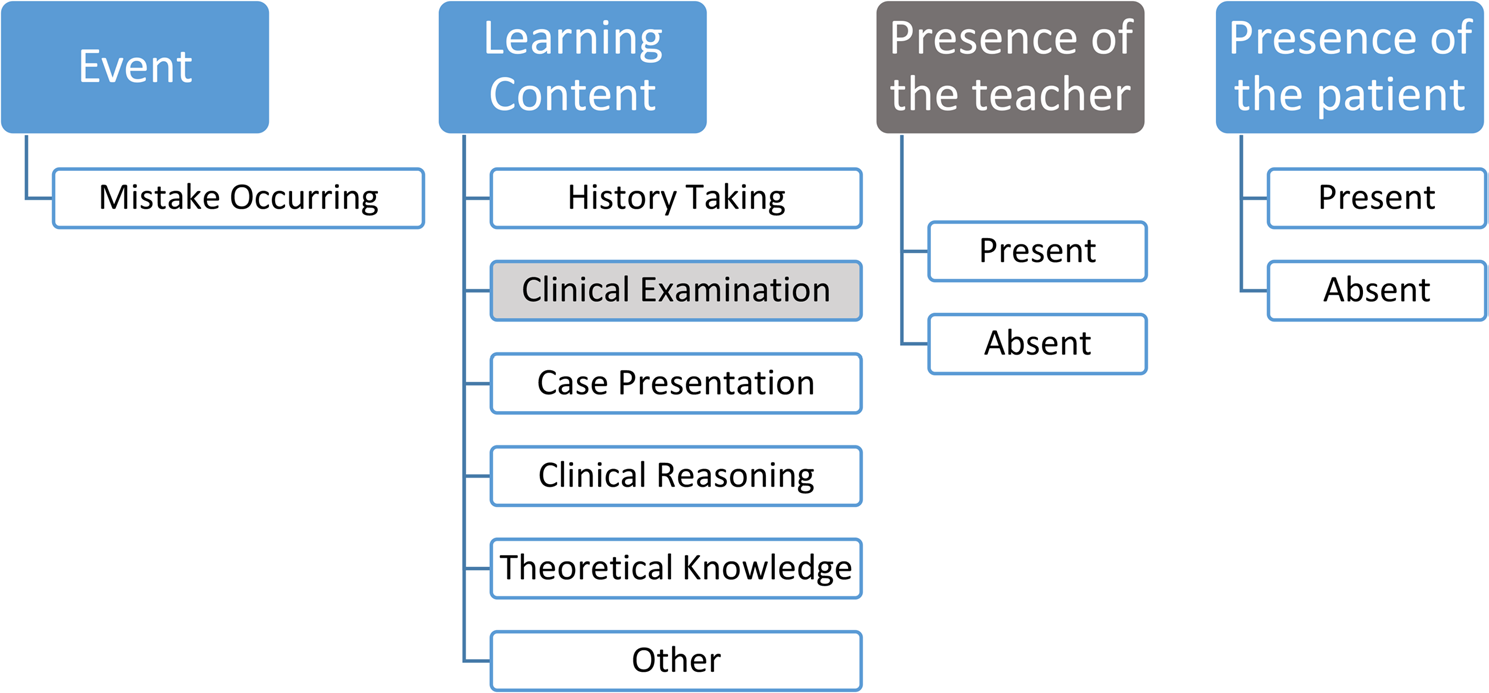
Categorical scheme for the event of an occurring student mistake (cf [47])

We drew upon pertinent literature [8, 56, 57] to code *learning content* (cf. Fig. 2) characteristically covered in bedside teaching sessions. We achieved very good [58] interrater reliability for *learning content* (0.83), *presence of the teacher* (0.99) and *presence of the patient* (> 0.99).

### Statistical analyses

We used *IBM SPSS* for *Windows* (version 26.0) to perform both descriptive and inferential statistical calculations. The significance threshold was set to *p* < 0.05. In the following section, our data are presented in terms of absolute and relative frequencies. The respective data were determined to be continuous but not normally distributed. Correlations were measured by Spearman’s rank correlation coefficient (RQ 2). To answer RQ 3, we focus on different categories of clinical teachers’ reactions to student mistakes: feedback, level of elaboration and time for correction of a mistake. The respective data had binary characteristics, and Mann‒Whitney U tests were applied to test for differences in central tendency in the teachers’ reactions to mistakes depending upon the presence and absence of the patient (RQ 3).

## Results

### RQ1: How is the learning content covered in bedside teaching related to the frequency and types of student mistakes?

The 36 BST sessions we filmed had a mean duration of 128.9 min (*SD* = 30.5 min); the shortest session lasted 79.9 min, and the longest session lasted 181.9 min, with an IQR of 57.0 min from 103.6 to 160.6 min, cf. overall duration in [49], Figure 3: This figure depicts the heterogeneity of the filmed BST sessions regarding overall duration and learning content, apparent from the strongly scattered data.

#### Learning content and student mistakes

We were able to assign a specific type of learning content from our categorial system to 81.4% of the overall BST time recorded. (18.6% of the time, the learning content was coded as “other”; cf. Fig. 2). Table 1 summarises the number of errors per hour stratified by learning content and error type. Overall, we counted an average of 4.8 errors per hour of BST. The highest error rates occurred during Clinical Examination, Case Presentation, and Theoretical Knowledge. Regarding error type, application errors (affecting both skills and knowledge) predominated during Clinical Examination and Case Presentation, whereas reproduction errors were most frequent during Theoretical Knowledge and Clinical Reasoning tasks.

Detailed distributions of error frequency by learning content and error type are provided in Table 1: Overall, we observed relatively high frequencies of student mistakes (of all types) in the context of the types of learning content: *clinical examination* (7.9 mistakes per hour on average), *case presentation* (6.4 mistakes per hour on average), *theoretical knowledge* (6.3 mistakes per hour on average), and clinical reasoning (5.1 mistakes per hour on average; cf. Table 1). Lower frequencies of student mistakes were observed in the context of *clinical examination* (3.6 *application mistakes [skills]* per hour), *clinical reasoning* (2.3 *reproduction mistakes* per hour) and *case presentation* (2.1 *application mistakes [skills]* per hour). The type of learning content in which we observed the highest frequency of student mistakes was *theoretical knowledge* (4.4 reproduction mistakes per hour), which, at the same time, was the type of learning content on which the smallest share of time was spent (cf [49]. ; *MN* = 7 min, *IQR* = 1–14 min).

**Table 1:** Frequency and type of student mistakes per hour during different types of learning content covered in BST

		Type of mistake per hour							Overall per hour
		Wrong Application of Skills	Wrong Application of Knowledge	Analytic Mistakes	Reproduction Mistakes	Other Mistakes	Mistakes related to Misinterpretation	Comprehension Mistakes	
Learning Content	CE	3.570	1.092	0.252	1.890	0.210	0.042	0.798	7.854
	CP	2.074	1.082	0.000	1.803	0.090	0.090	1.262	6.402
	CR	0.000	0.774	0.915	2.322	0.070	0.070	0.985	5.137
	HT	0.366	0.000	0.122	0.122	0.000	0.000	0.000	0.610
	TK	0.000	0.569	0.000	4.364	0.000	0.190	1.139	6.262
	OT	0.068	0.068	0.068	0.068	0.068	0.068	0.068	0.068
All Learning Contents per hour		1.448	0.672	0.259	1.577	0.090	0.052	0.711	4.809

*CE* Clinical Examination, *CP* Case Presentation, *CR* Clinical Reasoning, *HT* History Taking, *TK* Theoretical Knowledge, *OT* Other

### RQ2: influence of the presence of the clinical teacher on student mistakes during the clinical examination

Table 2 displays the descriptions of different types of student mistakes and the percentage of time the medical teacher was present during the clinical examination. Because clinical teachers frequently rotated between student sub-groups, teacher presence was rarely a binary state (entirely present vs. entirely absent) and is therefore calculated as the continuous percentage of time the teacher was present during the respective phase.

**Table 2:** Student mistakes and presence of the clinical teacher during the clinical examination in BST

	Mean	SD	Median	IQR	Min–Max
Clinical Examination duration (min)	26.6	21.7	22.1	13.0–35.6	3.4–117.8
Teacher presence (% of time)	72	42	100	38–100	0–100
Total Mistakes (count)	3.52	5,28	2	0–4	0–23
Reproduction Mistakes	0.93	1,62	0	0–2	0–6
Comprehension Mistakes	0.41	1,04	0	0–0	0–5
Wrong Application of Skills	1.59	2,71	0	0–3	0–14
Wrong Application of Knowledge	0.48	1,30	0	0–0	0–8
Analytic Mistakes	0.11	0,37	0	0–0	0–2

Sample: *N* = 54 clinical examinations

Owing to the large differences in the length of the clinical examination (from 3 to 118 min), we do not outline the absolute time but rather the percentage of time the medical teacher was present during the clinical examination. On average, the time dedicated to teaching, learning and practising the clinical examination was 27 min (*SD* = 22 min). In the BST sessions we filmed, the medical teacher was present during the clinical examination 72.00% of the time (min = 0.00%, max = 100.00% of the time, *IQR* = 39.25% − 100.00%). On average, 3.50 (*SD* = 5.20) student mistakes occurred during the clinical examination. As might be expected, the absolute number of mistakes was inherently linked to the duration of the examination; very brief practice phases (e.g., the minimum duration of roughly 3.5 min) naturally yielded zero observed mistakes, whereas longer examinations provided more opportunities for mistakes to occur and be documented.

### Correlations

We found positive correlations for all variables examined. Below, the effects, which have an uncorrected p-value < 0.05, are discussed. All correlations are shown in Table 3.

**Table 3:** Examining non-parametric correlations between teacher presence and student mistakes during the clinical examination

Spearman-Rho correlation coefficients: Teacher presence (percentage of time)									
	Teacher Presence	Mistakes in clinical examination	Reproduction Mistakes		Comprehension Mistakes		Wrong Application of Skills	Wrong Application of Knowledge	Analytic Mistakes
Correlation coefficient	1.000	0.483	0.401		0.282		0.252	0.235	0.237
Sig. (2-tailed)	.	< 0.001	0.003		0.039		0.066	0.087	0.084
N	54	54	54		54		54	54	54

We performed non-parametric correlation analyses using Spearman’s rank correlation coefficient, the results of which are presented in Table 3: We found a significant positive correlation between the presence of the clinical teacher and the number of student mistakes observed in general and reproduction mistakes in particular, meaning that more mistakes were recorded when teachers were present for larger portions of the session. Importantly, given our correction-based operationalisation of mistakes, this likely reflects increased *detection and verbal addressing* of mistakes rather than a higher underlying error rate. Both correlations are significant after correction for multiple testing. This direct relationship, and the question of *when* these mistakes occurred, is largely explained by our methodology: because we coded a mistake based on the overt occurrence of a correction, mistakes that happened when the teacher was absent largely went unnoticed and unrecorded (unless a peer intervened). Therefore, the recorded mistakes in our dataset predominantly occurred in the presence of the teacher. We strongly suspect that during unsupervised phases of the clinical examination, many additional mistakes occurred, but they remained invisible to our analysis because there was no teacher present to identify and address them. We did not find a significant correlation between the presence of the teacher and the occurrence of any other mistakes.

### RQ3: influence of the presence of the patient on clinical teachers’ reactions to student mistakes

Finally, we investigated whether the reactions of the clinical teachers to student mistakes changed depending upon the presence or absence of the patient (cf. Tables 4 and 5). We coded the presence of the patient by selecting *patient room* for *location*, cf. our categorial system: [49].

**Table 4:** Frequencies of different types of clinical teachers’ reactions to student mistakes (feedback; level of elaboration and time for correction) in relation to the presence vs. absence of a patient in BST

Feedback	Explicit rejection	Further inquiry	Mean Rank	Rank Sum
Patient absent	166 *(64.80%)*	90 *(35.20%)*	181.39	46436.00
Patient present	65 *(56.00%)*	51 *(44.00%)*	197.78	22942.00
Level of elaboration	Low	High	Mean Rank	Rank Sum
Patient absent	114 *(44.50%)*	142 *(55.50%)*	187.17	47916.00
Patient present	53 *(45.70%)*	63 *(54.30%)*	185.02	21462.00
Time for correction	No	Yes	Mean Rank	Rank Sum
Patient absent	118 *(46.10%)*	138 *(53.90%)*	184.77	47300.00
Patient present	50 *(43.10%)*	66 *(56.90%)*	190.33	22078.00

**Table 5:** Patient presence and teacher reactions: inferential statistical comparisons

	Feedback	Level of Elaboration	Time for correction
Mann‒Whitney-U Test	13,540	14,676	14,404
Wilcoxon W	46,436	21,462	47,300
Z	-1.620	-0.208	-0.536
Asymptotic Significance (2-tailed)	0.105	0.835	0.592

We found no significant differences regarding teachers’ reactions to student mistakes, depending upon the presence or absence of a patient. Although the clinical teachers in our study reacted less frequently with explicit rejection to student mistakes when a patient was present (56.00% compared with 64.80% when no patient was present), the Mann‒Whitney U test did not reach statistical significance. Additionally, the teachers did not significantly modify their levels of elaboration or provide more time for correction after a mistake, depending upon the presence of a patient.

## Discussion

This study identified correlations between learning content, teacher presence, and the occurrence of student mistakes in BST. Furthermore, we analysed how clinical teachers reacted to these mistakes, specifically examining whether patient presence influenced their responses. Regarding the patient’s role, we found no statistically significant differences in teachers’ reactions to student mistakes - including their use of explicit rejection, elaboration, or time provided for correction - based on the patient’s presence or absence.

In our analysis, we focused on the characteristic educational objectives of BST as reported in the pertinent literature, namely, history taking, clinical examination, case presentation, clinical reasoning and theoretical knowledge [14, 15, 46], and investigated the frequency and types of student mistakes occurring within these contexts. Overall, during the BST sessions observed, almost five student mistakes occurred per hour on average, which underlines that clinical teachers should not only correct but also actively address these mistakes and make didactic use of them. The sheer frequency of student mistakes suggests that instruction in the context of BST will be more effective if teachers handle student mistakes in didactically productive ways to address gaps in student knowledge and skills [59].

When we looked closer into how many mistakes occurred in different types of learning content covered in BST, we found significant differences. To aid clarity in interpreting these findings, we briefly recall our operational definitions: while “mistakes” and “errors” are used interchangeably as general terms, our coding framework delineates specific sub-types, such as “reproduction mistakes” (failures in remembering basic knowledge) and “wrong application” (incorrect procedural execution). While the teaching of theoretical knowledge played a minor role in terms of time allocation within the BST sessions we filmed, many mistakes occurred in this context, particularly reproduction mistakes. These are mistakes that occur when remembering or retrieving content that has been previously learned [51], such as basic medical knowledge. This pattern might suggest challenges with retaining foundational knowledge or accessing it under the cognitive load inherent in clinical scenarios. Furthermore, we also observe many reproduction mistakes in the context of the learning content clinical reasoning. These recurring reproduction mistakes could also indicate deeper, underlying misconceptions - flawed or incomplete understandings that resist simple correction. Identifying these specific mistakes requires more than surface-level correction; diagnostic approaches such as targeted questioning or analysing error patterns are needed. However, during clinical examination and case presentations, we primarily observed the wrong application of skills-type mistakes. These specific “wrong application” mistakes relate more directly to procedural execution or integrating skills in practice. This distinction highlights the need for targeted, observation-based feedback focused on procedural aspects, which inherently relies on direct supervision.

A crucial framing for the following interpretations of our findings is that our data reflect detected and corrected mistakes rather than the total number of mistakes that actually occurred. On this basis, we focused the learning content physical examination, as it is argued that the bedside is the ideal place for teaching such clinical skills [23, 60]. In our sample, we found that clinical examination was performed in almost all (but three) sessions. However, on average, clinical teachers supervised the students only 72% of the time they were practising clinical examinations. Hence, 28% of the time, potential mistakes during the performance of the examination may have gone unnoticed - if there was no fellow student who recognised and pointed out the mistake. This lack of consistent supervision is a significant concern, as it represents missed learning opportunities and carries the risk of students reinforcing incorrect techniques through unguided practice. Because clinical teachers cannot simultaneously supervise multiple small groups, they should explicitly instruct students to peer-monitor, identify mistakes, and consult the teacher when unsure. The observed lack of direct observation remains a critical issue, aligning with previous literature [4, 60–62]. In our categorical scheme for the event of an occurring mistake, we define the code ‘mistake’ on the basis of a correction made by the teacher or another student [50]. Thus, we might have underestimated the actual mistake rate during unsupervised practice. However, how effectively undergraduate medical students can detect and correct their fellow students’ mistakes, given their limited clinical experience, remains an open question. Effective peer feedback requires goals, training, and a supportive environment, which may not always be present. As discussed above, we found a significant positive correlation between the presence of the clinical teacher and the number of student mistakes observed in general and reproduction mistakes in particular. This pattern is strongly shaped by our operational definition, which ties the identification of a mistake to an observable correction; however, we argue that during the clinical examination in undergraduate BST, mistakes occur, which may go unnoticed if the student group is unsupervised but are more likely to be identified and addressed by a present teacher. Consequently, our findings do not allow conclusions about whether students make more mistakes in the presence of teachers; rather, they indicate that mistakes are more likely to be identified and addressed in pedagogically productive ways if they occur under supervision by a clinical teacher.

Based on our results, further critical questions arise regarding the degree to which mistakes made during BST seminars are corrected, since students may find themselves mostly relying on the clinical expertise of their fellow students. The results of RQ2 confirm this, as only approximately two-thirds of the time dedicated to the “teaching” physical examination involved the presence of a teacher. These findings are congruent with the pertinent literature: Haring et al. noted that direct observation of the students’ interactions with patients, particularly their physical examination skills, appears to not be standard practice [61–63], whereas Rousseau et al. [4, 22] argued that there is a “lack of direct observation of trainees in performing [physical examination] skills”. Various reasons for this lack of observation during the clinical examination have been proposed in recent years, with the “hospital environment and its chaotic nature, time constraints and conflicting responsibilities” [4] having been identified as the most important barriers [23, 64, 65]. These systemic pressures must be evaluated and taken into account when planning BST sessions.

While it might well be that teacher presence led to an increased rate of mistakes being recognized and pointed out, also other explanations are possible. First, maybe teachers were more present during phases where students were engaged with cognitively or procedurally more challenging tasks on purpose - so they could help students better or correct more mistakes. Likewise, students may have unconsciously behaved differently when they noticed the clinical teacher being around - either because they felt more observed and controlled, or because they felt more guided by and more experienced colleague. Investigating such hypotheses is a promising goal for future research. Some pertinent research has shown that teachers’ error management behaviour is likely to influence students’ attitudes towards learning from mistakes [47, 66], as “teachers’ maladaptive ways of handling’ students’ mistakes are likely to increase students’ fear of failure and may foster maladaptive motivational patterns, such as […] experiencing generalized negative emotions” in the context of failures [47]. Effectively leveraging mistakes requires fostering an error management culture, where errors are viewed as learning opportunities rather than solely as incidents to be avoided. This culture encourages open discussion and analysis of errors for improvement. Central to this is psychological safety [67] - the shared belief that the environment is safe for interpersonal risk-taking, such as admitting errors or asking questions, without fear of negative consequences. When learners do not feel psychologically safe, they may hesitate to engage in the various behaviours needed to learn from mistakes [59]. However, when discussing student mistakes and teachers’ reactions in BST, it is important to keep in mind the presence of the patient as a third party. While mistakes hold the potential for students to feel embarrassed in front of a patient, they may also be sensitive situations for the doctor-patient relationship [23]. In that sense, the patient’s presence creates a complex triadic interaction. In another paper by our research group discussing student mistakes in BST, we hypothesized that “doctors are aware of this situation and adjust their response in front of the patient”, calling for “further research on […] how teachers’ reactions change depending upon the presence and absence of patients” [22].

In the present study, we did not observe that clinical teachers’ reactions to student mistakes changed depending upon the presence or absence of a patient. Teachers did not significantly modify their levels of elaboration, the directness of negative feedback, or the time they provided for correction of student mistakes, depending upon whether a patient was present. As our results do not warrant any interpretation of how patient presence shapes teachers’ behaviour in BST, the following reflections are merely tentative and dedicated to stimulate further theoretical and empirical inquiry: While this finding could point towards a lack of awareness regarding patient presence versus absence, attributing this null finding solely to unawareness is likely an oversimplification. Instead, this consistency in feedback behaviour, regardless of patient presence, may reflect ingrained pedagogical norms and the professional habitus of clinical educators. However, our data do not allow us to directly test this explanation. Teachers may prioritize standardized routines of immediate error correction and medical accuracy over navigating the complex social nuances of the patient’s room. Furthermore, structural constraints - such as the chaotic hospital environment, significant time limitations, and conflicting responsibilities - impose a high cognitive load that may limit a clinical teacher’s capacity to consciously adapt their pedagogical style on the fly. It is also plausible that educators operate under the assumption that authentic clinical training necessitates transparent correction, thereby maintaining their standard instructional approach regardless of the audience. Additionally, the clinical teachers in our sample may possess a high level of comfort and experience with bedside teaching, allowing them to effortlessly navigate the patient-student-teacher triad without needing to alter their didactic strategies. Finally, the observational nature of the research itself must be considered. Knowing they were being video recorded may have prompted tutors to consciously or subconsciously adopt a more standardized, ‘professional’ approach to error correction across all situations (i.e., the Hawthorne effect). While assessing this dynamic covertly would theoretically eliminate such an observer effect, covert observation is ethically precluded in this clinical setting. Future research should focus on the degree of clinical teachers’ sensitivity in this respect because the opportunity for better learning from mistakes might be deferred if not handled with specific strategies, such as briefly acknowledging the mistake, using guiding questions, or explicitly ‘bookmarking’ it for later debriefing.

Whether clinical teachers succeed in tapping the learning potential of mistakes depends on how they handle them, i.e., how they are reported back to the students and if and how they are further discussed. Effective feedback is crucial in this respect; it should be specific, descriptive, actionable, timely, balanced, objective, and learner-engaging. Models such as Pendleton’s rules, Ask-Tell-Ask, or ARCH can provide structure [68]. However, the effectiveness of any feedback hinges on the underlying psychological safety, as was already discussed previously. In our study, we analysed how teachers handle student mistakes in bedside teaching. The questions of how well students actually learn from these mistakes and how the way mistakes are handled by clinical teachers influences students’ learning outcomes remain open to be addressed in further research [54].

Furthermore, the finding that seminars filmed were sometimes shorter than the scheduled 180 min suggests potential unused time. This time could be strategically employed for active learning, specifically targeting common mistakes or misconceptions identified during the session. Strategies such as brief diagnostic probes, think-pair-sharing activities, or focused retrieval practices could transform these minutes into valuable, targeted learning opportunities, enhancing the efficiency of BST [69].

To increase the practical relevance of our study’s implications, some rather concrete points for faculty development can be derived: We found that teacher presence varied substantially during skill‑based activities - which might increase between-student heterogeneity and lead to teachers’ missing valuable instructional opportunities. As continuous supervision is not always feasible, clinical teachers could benefit from guidance on how they can plan their students’ supervision more intentionally across different phases of BST and across different smaller student groups. Crucially, faculty development programs must acknowledge the structural constraints and ingrained pedagogical norms that influence clinical teaching. To this end, short, structured supervision models should be favoured, like structured check‑ins or mini‑debriefings, which can be tailored to focus the identification of mistakes that might otherwise remain unnoticed without adding excessive cognitive load on the instructor. Such targeted, low‑threshold strategies align well with the environmental challenges observed in our study and may help clinical teachers make more consistent use of the learning opportunities inherent in BST while working within their existing institutional constraints.

### Limitations

Regarding the limitations of the present study, our operational definition of a mistake leaves open the question of the extent to which unaddressed mistakes occur in BST - meaning errors that go unnoticed by both the clinical teacher and the students. Investigating this was not feasible in the context of the present study, as it would have required extensive additional video coding by subject matter experts across the various clinical disciplines involved. This represents an important limitation of our operationalisation, as it likely leads to a systematic underestimation of mistakes, particularly during unsupervised phases. Consequently, the frequencies reported here should be interpreted as conservative estimates of observable errors rather than comprehensive measures of error occurrence. We could not identify any existing research in the medical education literature that systematically focuses the factual accuracy of clinical teaching content, either during bedside teaching or in other instructional formats. Furthermore, while we recorded the overall percentage of teacher presence per clinical examination, our methodology did not allow for a timestamp-level mapping of individual mistakes to the exact moment of teacher presence or absence. Therefore, we cannot empirically isolate the exact number of mistakes that occurred specifically with or without the tutor present, which limits our ability to draw definitive conclusions regarding this specific micro-dynamic. Additionally, because our study relies on cross-sectional observations of isolated BST sessions with different groups of students each time, we were unable to track individual learning trajectories. Consequently, we cannot determine longitudinally whether students’ mistakes decreased, were sustainably corrected, or if new mistakes arose over time. Future research should consider longitudinal designs to explore how error patterns evolve as students advance through their clinical training. Finally, the observed nature of the research itself must be considered as a limitation. Knowing they were being video recorded may have prompted the clinical teachers to consciously or subconsciously adopt a more standardized, ‘professional’ approach to error correction across all situations, regardless of patient presence (i.e., the Hawthorne effect). While assessing this dynamic covertly would theoretically eliminate such an observer effect, covert observation is ethically precluded in this clinical and educational setting.

## Conclusions

In sum, student mistakes are frequent in BST and represent significant learning opportunities. Capitalising on these mistakes requires teachers to move beyond straightforward correction of mistakes towards fostering a psychologically safe environment where mistakes can be openly discussed and analysed. Addressing the challenge of inconsistent supervision, particularly during skill-based activities such as clinical examination, is crucial in our view. Teachers also need to be supported in developing good strategies to provide effective, elaborate feedback and navigate the complexities of the patient’s presence, perhaps by encouraging reflective practices that challenge traditional pedagogical habits. Recognising patterns in student mistakes, diagnosing underlying misconceptions, and potentially utilising available time for targeted active learning are key areas for improving the pedagogical effectiveness of BST. Future research should continue to explore student perspectives on errors and feedback, evaluate interventions aimed at improving error management and feedback practices, and investigate the long-term impact of psychologically safe learning environments.

## Data Availability

All data (except the original video recordings) and materials are available upon direct request to the corresponding author: [martin.gartmeier@tum.de] .

## Footnotes

1 Exhaustive-disjunctive categorial system: includes all possibilities (that have not been ruled out). Example for the video code *social structure*: *plenary setting*, *small group setting* or *other*.

## Abbreviations

BST: Bedside Teaching
IQR: Interquartile range
MN: Mean
RQ: Research Question
SD: Standard deviation

## References

[1] Ahmed M-B. What is happening to bedside clinical teaching? Med Educ. 2002;36:1185–8. 10.1046/j.1365-2923.2002.01372.x. 12472754 10.1046/j.1365-2923.2002.01372.x

[2] Rizan C, Elsey C, Lemon T, Grant A, Monrouxe LV. Feedback in action within bedside teaching encounters: a video ethnographic study. Med Educ. 2014;48:902–20. 10.1111/medu.12498. 25113117 10.1111/medu.12498

[3] Dybowski C, Harendza S. Bedside Teaching: general and discipline-specific teacher characteristics, criteria for patient selection and difficulties. GMS Z Med Ausbild. 2013;30:Doc23. 10.3205/zma000866. 23737920 10.3205/zma000866 PMC3671319

[4] Rousseau M, Könings KD, Touchie C. Overcoming the barriers of teaching physical examination at the bedside: more than just curriculum design. BMC Med Educ. 2018;18:302. 10.1186/s12909-018-1403-z. 30537960 10.1186/s12909-018-1403-z PMC6288852

[5] Peters M, Cate O. ten. Bedside teaching in medical education: a literature review. Perspect Med Educ. 2014;3:76–88. 10.1007/s40037-013-0083-y. 10.1007/s40037-013-0083-y PMC3976479 24049043

[6] Nair BR, Coughlan JL, Hensley MJ. Impediments to bed-side teaching. Med Educ. 1998;32:159–62. 10.1046/j.1365-2923.1998.00185.x. 9743767 10.1046/j.1365-2923.1998.00185.x

[7] Dewey JJ, Cho TA. Bedside Teaching in Neurology. Semin Neurol. 2018;38:441–8. 10.1055/s-0038-1666984. 30125898 10.1055/s-0038-1666984

[8] Gonzalo JD, Heist BS, Duffy BL, Dyrbye L, Fagan MJ, Ferenchick GS, et al. The value of bedside rounds: a multicenter qualitative study. Teach Learn Med. 2013;25:326–33. 10.1080/10401334.2013.830514. 24112202 10.1080/10401334.2013.830514

[9] Wenrich MD, Jackson MB, Ajam KS, Wolfhagen IH, Ramsey PG, Scherpbier AJ. Teachers as learners: the effect of bedside teaching on the clinical skills of clinician-teachers. Acad Med. 2011;86:846–52. 10.1097/ACM.0b013e31821db1bc. 21617505 10.1097/ACM.0b013e31821db1bc

[10] Alpert JS. Some thoughts on bedside teaching. Am J Med. 2009;122:203–4. 10.1016/j.amjmed.2008.10.024. 19272476 10.1016/j.amjmed.2008.10.024

[11] Sultan AS. Bedside teaching: An indispensible tool for enhancing the clinical skills of undergraduate medical students. J Pak Med Assoc. 2019;69:235–40. 30804590

[12] Gonzalo JD, Masters PA, Simons RJ, Chuang CH. Attending rounds and bedside case presentations: medical student and medicine resident experiences and attitudes. Teach Learn Med. 2009;21:105–10. 10.1080/10401330902791156. 19330687 10.1080/10401330902791156 PMC2696474

[13] Raupach T, Anders S, Pukrop T, Hasenfuss G, Harendza S. Effects of minimally invasive curricular surgery - a pilot intervention study to improve the quality of bedside teaching in medical education. Med Teach. 2009;31:e425–30. 10.1080/01421590902845865. 19811179 10.1080/01421590902845865

[14] Al-Swailmi FK, Khan IA, Mehmood Y, Al-Enazi SA, Alrowaili M, Al-Enazi MM. Students’ perspective of bedside teaching: A qualitative study. Pak J Med Sci. 2016;32:351–5. 10.12669/pjms.322.9194. 27182238 10.12669/pjms.322.9194 PMC4859021

[15] Dreiling K, Montano D, Poinstingl H, Müller T, Schiekirka-Schwake S, Anders S, et al. Evaluation in undergraduate medical education: Conceptualizing and validating a novel questionnaire for assessing the quality of bedside teaching. Med Teach. 2017;39:820–7. 10.1080/0142159X.2017.1324136. 28532203 10.1080/0142159X.2017.1324136

[16] Rees CE, Ajjawi R, Monrouxe LV. The construction of power in family medicine bedside teaching: a video observation study. Med Educ. 2013;47:154–65. 10.1111/medu.12055. 23323654 10.1111/medu.12055

[17] Aldeen AZ, Gisondi MA. Bedside teaching in the emergency department. Acad Emerg Med. 2006;13:860–6. 10.1197/j.aem.2006.03.557. 16766739 10.1197/j.aem.2006.03.557

[18] Gonzalo JD, Chuang CH, Huang G, Smith C. The return of bedside rounds: an educational intervention. J Gen Intern Med. 2010;25:792–8. 10.1007/s11606-010-1344-7. 20386997 10.1007/s11606-010-1344-7 PMC2896611

[19] Nair BR, Coughlan JL, Hensley MJ. Student and patient perspectives on bedside teaching. Med Educ. 1997;31:341–6. 10.1046/j.1365-2923.1997.00673.x. 9488854 10.1046/j.1365-2923.1997.00673.x

[20] Qureshi Z, Maxwell S. Has bedside teaching had its day? Adv Health Sci Educ. 2012;17:301–4. 10.1007/s10459-011-9308-1. 10.1007/s10459-011-9308-1 21681592

[21] Carraccio CL, Benson BJ, Nixon LJ, Derstine PL. From the educational bench to the clinical bedside: translating the Dreyfus developmental model to the learning of clinical skills. Acad Med. 2008;83:761–7. 10.1097/ACM.0b013e31817eb632. 18667892 10.1097/ACM.0b013e31817eb632

[22] Rubisch HPK, Blaschke A-L, Berberat PO, Fuetterer CS, Haller B, Gartmeier M. Student mistakes and teacher reactions in bedside teaching. Adv Health Sci Educ. 2023;28:1523–56. 10.1007/s10459-023-10233-y. 10.1007/s10459-023-10233-y PMC10174607 37170035

[23] Williams KN, Ramani S, Fraser B, Orlander JD. Improving bedside teaching: findings from a focus group study of learners. Acad Med. 2008;83:257–64. 10.1097/ACM.0b013e3181637f3e. 18316871 10.1097/ACM.0b013e3181637f3e

[24] Salam A, Siraj HH, Mohamad N, Das S, Rabeya Y. Bedside teaching in undergraduate medical education: issues, strategies, and new models for better preparation of new generation doctors. Iran J Med Sci. 2011;36:1–6. PMC3559110 23365470

[25] Pickles R. Bedside clinical teaching: Arresting the decline. Arch Med Health Sci. 2020;8:9. 10.4103/amhs.amhs_25_20.

[26] Faustinella F, Jacobs RJ. The decline of clinical skills: a challenge for medical schools. Int J Med Educ. 2018;9:195–7. 10.5116/ijme.5b3f.9fb3. 30007951 10.5116/ijme.5b3f.9fb3 PMC6129153

[27] van Dam M, Ramani S, ten Cate O. Breathing life into bedside teaching in the era of COVID-19. Med Teach. 2020;42:1310–2. 10.1080/0142159X.2020.1798368. 32726155 10.1080/0142159X.2020.1798368

[28] Carlos WG, Kritek PA, Clay AS, Luks AM, Thomson CC. Teaching at the Bedside. Maximal Impact in Minimal Time. Annals Am Thorac Soc. 2016;13:545–8. 10.1513/AnnalsATS.201601-018AS. 10.1513/AnnalsATS.201601-018AS 26845234

[29] Afifi L, Shinkai K. Optimizing education on the inpatient dermatology consultative service. Semin Cutan Med Surg. 2017;36:28–34. 10.12788/j.sder.2017.003. 28247873 10.12788/j.sder.2017.003

[30] Janicik RW, Fletcher KE. Teaching at the bedside: a new model. Med Teach. 2003;25:127–30. 10.1080/0142159031000092490. 12745518 10.1080/0142159031000092490

[31] LaCombe MA. On bedside teaching. Ann Intern Med. 1997;126:217–20. 10.7326/0003-4819-126-3-199702010-00007. 9027273 10.7326/0003-4819-126-3-199702010-00007

[32] Waydhas C, Taeger G, Zettl R, Oberbeck R, Nast-Kolb D. Improved student preparation from implementing active learning sessions and a standardized curriculum in the surgical examination course. Med Teach. 2004;26:621–4. 10.1080/01421590400019526. 15763852 10.1080/01421590400019526

[33] Verghese A, Brady E, Kapur CC, Horwitz RI. The bedside evaluation: ritual and reason. Ann Intern Med. 2011;155:550–3. 10.7326/0003-4819-155-8-201110180-00013. 22007047 10.7326/0003-4819-155-8-201110180-00013

[34] Hascher T, Hagenauer G. Lernen aus Fehlern. In: Spiel C, Schober B, Wagner P, Reimann R, editors. Bildungspsychologie; 2010. p. 377–81.

[35] Stillman PL, Regan MB, Swanson DB, Case S, McCahan J, Feinblatt J, et al. An assessment of the clinical skills of fourth-year students at four New England medical schools. Acad Med. 1990;65:320–6. 10.1097/00001888-199005000-00013. 2337437 10.1097/00001888-199005000-00013

[36] Braun LT, Zwaan L, Kiesewetter J, Fischer MR, Schmidmaier R. Diagnostic errors by medical students: results of a prospective qualitative study. BMC Med Educ. 2017;17:191. 10.1186/s12909-017-1044-7. 29121903 10.1186/s12909-017-1044-7 PMC5679151

[37] Friedman MH, Connell KJ, Olthoff AJ, Sinacore JM, Bordage G. Medical student errors in making a diagnosis. Acad Med. 1998;73:S19–21. 10.1097/00001888-199810000-00033. 9795640 10.1097/00001888-199810000-00033

[38] Clifford MM. Risk Taking: Theoretical, Empirical, and Educational Considerations. Educational Psychol. 1991;26:263–97. 10.1080/00461520.1991.9653135.

[39] Dresel M, Schober B, Ziegler A, Grassinger R, Steuer G. Affektiv-motivational adaptive und handlungsadaptive Reaktionen auf Fehler im Lernprozess*. Z für Pädagogische Psychologie. 2013;27:255–71. 10.1024/1010-0652/a000111.

[40] Weinert FZ. Aus Fehlern lernen und Fehler vermeiden lernen. In: Fehlerwelten: VS Verlag für Sozialwissenschaften, Wiesbaden;; 1999. pp. 101–9. 10.1007/978-3-663-07878-4_5.

[41] Oser F, Spychiger M. Lernen ist schmerzhaft: Zur Theorie des negativen Wissens und zur Praxis der Fehlerkultur. Weinheim, Basel: Beltz; 2005.

[42] Doshi M, Brown N. Whys and hows of patient-based teaching. Adv psychiatr treat. 2005;11:223–31. 10.1192/apt.11.3.223.

[43] Bauer J. (2008). Learning from errors at work. Studies on nurses’ engagement in error-related learning activities. Dissertation, Universität Regensburg 2008. https://epub.uni-regensburg.de/10748/1/diss_veroeff_endversion.pdf.

[44] Heid H. Autorität — Über die Verwandlung von Fehlern in Verfehlungen. In: Fehlerwelten: VS Verlag für Sozialwissenschaften, Wiesbaden;; 1999. pp. 129–36. 10.1007/978-3-663-07878-4_7.

[45] Anderson LW, David R, Krathwohl, Bloom BS. A Taxonomy for Learning, Teaching, and Assessing. a Revision of Bloom’s Taxonomy of Educational Objectives. London: Pearson; 2001.

[46] Gonzalo JD, Heist BS, Duffy BL, Dyrbye L, Fagan MJ, Ferenchick G, et al. The art of bedside rounds: a multi-center qualitative study of strategies used by experienced bedside teachers. J Gen Intern Med. 2013;28:412–20. 10.1007/s11606-012-2259-2. 23129164 10.1007/s11606-012-2259-2 PMC3579967

[47] Tulis M. Error management behavior in classrooms: Teachers’ responses to student mistakes. Teach Teacher Educ. 2013;33:56–68. 10.1016/j.tate.2013.02.003.

[48] McDermott A. Championing Mistakes: Reclaiming the Safe Learning Environment for Family-Centered Bedside Rounds. J Grad Med Educ. 2017;9:257. 10.4300/JGME-D-16-00664.1. 28439368 10.4300/JGME-D-16-00664.1 PMC5398155

[49] Blaschke A-L, Rubisch HPK, Schindler A-K, Berberat PO, Gartmeier M. How is modern bedside teaching structured? A video analysis of learning content, social and spatial structures. BMC Med Educ. 2022;22:790. 10.1186/s12909-022-03855-0. 36380308 10.1186/s12909-022-03855-0 PMC9664733

[50] Seidel T. How to run a video study: Technical report of the IPN Video Study. Münster: Waxmann; 2005.

[51] Mindnich A, Wuttke E, Seifried J. Aus Fehlern wird man klug? Eine Pilotstudie zur Typisierung von Fehlern und Fehlersituationen. In: Lankes E-M, editor. Pädagogische Professionalität als Gegenstand empirischer Forschung. Münster: Waxmann. 2008;153–64.

[52] Bloom BS. Taxonomy of educational objectives: The classification of educational goals. New York: Longmans, Green; 1956.

[53] Wuttke E. Unterrichtskommunikation und Wissenserwerb: Zum Einfluss von Kommunikation auf den Prozess der Wissensgenerierung. Frankfurt am Main: Lang; 2005.

[54] Wuttke E. Umgang mit Fehlern und Ungewißheit im Unterricht: Entwicklung eines Beobachtungsinstruments und erste empirische Befunde. In: Gläser-Zikuda M, Seifried J, editors. Lehrerexpertise: Analyse und Bedeutung unterrichtlichen Handelns. Münster: Waxmann; 2008. pp. 91–111.

[55] Crespo S. Praising and correcting: prospective teachers investigate their teacherly talk. Teach Teacher Educ. 2002;18:739–58. 10.1016/S0742-051X(02)00031-8.

[56] Jünger J, Schäfer S, Roth C, Schellberg D, Friedman Ben-David M, Nikendei C. Effects of basic clinical skills training on objective structured clinical examination performance. Med Educ. 2005;39:1015–20. 10.1111/j.1365-2929.2005.02266.x. 16178828 10.1111/j.1365-2929.2005.02266.x

[57] Baker RC, Spence RA, Boohan M, Dorman A, Stevenson M, Kirk SJ, McGlade K. A novel approach to improve undergraduate surgical teaching. Ulster Med J. 2015;84:30–6. 25964701 PMC4330803

[58] Landis JR, Koch GG. The Measurement of Observer Agreement for Categorical Data. Biometrics. 1977;33:159. 10.2307/2529310. 843571

[59] Said Said Elshama. How to Use and Apply Assessment Tools in Medical Education? 2020. 10.5281/ZENODO.3978444.

[60] Scager K, Boonstra J, Peeters T, Vulperhorst J, Wiegant F. Collaborative Learning in Higher Education: Evoking Positive Interdependence. CBE Life Sci Educ. 2016. 10.1187/cbe.16-07-0219. 10.1187/cbe.16-07-0219 PMC5132366 27909019

[61] Haring CM, Cools BM, van der Meer JW, Postma CT. Student performance of the general physical examination in internal medicine: an observational study. BMC Med Educ. 2014;14:73. 10.1186/1472-6920-14-73. 24712683 10.1186/1472-6920-14-73 PMC4233641

[62] Burdick WP, Schoffstall J. Observation of emergency medicine residents at the bedside: how often does it happen? Acad Emerg Med. 1995;2:909–13. 10.1111/j.1553-2712.1995.tb03108.x. 8542492 10.1111/j.1553-2712.1995.tb03108.x

[63] Pulito AR, Donnelly MB, Plymale M, Mentzer RM. What do faculty observe of medical students’ clinical performance? Teach Learn Med. 2006;18:99–104. 10.1207/s15328015tlm1802_2. 16626266 10.1207/s15328015tlm1802_2

[64] Gonzalo JD, Heist BS, Duffy BL, Dyrbye L, Fagan MJ, Ferenchick G, et al. Identifying and overcoming the barriers to bedside rounds: a multicenter qualitative study. Acad Med. 2014;89:326–34. 10.1097/ACM.0000000000000100. 24362381 10.1097/ACM.0000000000000100

[65] Ramani S, Orlander JD, Strunin L, Barber TW. Whither bedside teaching? A focus-group study of clinical teachers. Acad Med. 2003;78:384–90. 10.1097/00001888-200304000-00014. 12691971 10.1097/00001888-200304000-00014

[66] Steuer G, Rosentritt-Brunn G, Dresel M. Dealing with errors in mathematics classrooms: Structure and relevance of perceived error climate. Contemp Educ Psychol. 2013;38:196–210. 10.1016/j.cedpsych.2013.03.002.

[67] Edmondson AC, Higgins M, Singer S, Weiner J. Understanding Psychological Safety in Health Care and Education Organizations: A Comparative Perspective. Res Hum Dev. 2016;13:65–83. 10.1080/15427609.2016.1141280.

[68] van de Ridder JMM. Pendleton’s Rules: A Mini Review of a Feedback Method. AJBSR. 2023;19:19–21. 10.34297/AJBSR.2023.19.002542.

[69] Duran MJ, Aciego JJ, Gonzalez-Prieto I, Carrillo-Rios J, Gonzalez-Prieto A, Claros-Colome A. A Gamified Active-Learning Proposal for Higher-Education Heterogeneous STEM Courses. Educ Sci. 2025;15:10. 10.3390/educsci15010010.

